# Activation state-dependent interaction between Gα_q_ subunits and the Fhit tumor suppressor

**DOI:** 10.1186/1478-811X-11-59

**Published:** 2013-08-15

**Authors:** Hao Zuo, Grace P W Chan, Jing Zhu, Wendy W S Yeung, Anthony S L Chan, Hermann Ammer, Yung H Wong

**Affiliations:** 1 Division of Life Sciences, Biotechnology Research Institute, Hong Kong University of Science and Technology, Clear Water Bay, Kowloon, Hong Kong; 2 Department of Veterinary Sciences, University of Munich, Munich, Germany; 3 State Key Laboratory of Molecular Neuroscience, Molecular Neuroscience Center, Hong Kong University of Science and Technology, Clear Water Bay, Kowloon, Hong Kong

**Keywords:** Fhit, G protein, Phospholipase Cβ, Src, Tumor suppression

## Abstract

**Background:**

The *FHIT* tumor suppressor gene is arguably the most commonly altered gene in cancer since it is inactivated in about 60% of human tumors. The Fhit protein is a member of the ubiquitous histidine triad proteins which hydrolyze dinucleoside polyphosphates such as Ap_3_A. Despite the fact that Fhit functions as a tumor suppressor, the pathway through which Fhit inhibits growth of cancer cells remains largely unknown. Phosphorylation by Src tyrosine kinases provides a linkage between Fhit and growth factor signaling. Since many G proteins can regulate cell proliferation through multiple signaling components including Src, we explored the relationship between Gα subunits and Fhit.

**Results:**

Several members of the Gα_q_ subfamily (Gα_16_, Gα_14_, and Gα_q_) were found to co-immunoprecipitate with Fhit in their GTP-bound active state in HEK293 cells. The binding of activated Gα_q_ members to Fhit appeared to be direct and was detectable in native DLD-1 colon carcinoma cells. The use of Gα_16/z_ chimeras further enabled the mapping of the Fhit-interacting domain to the α2-β4 region of Gα_16_. However, Gα_q_/Fhit did not affect either Ap_3_A binding and hydrolysis by Fhit, or the ability of Gα_q/16_ to regulate downstream effectors including phospholipase Cβ, Ras, ERK, STAT3, and IKK. Functional mutants of Fhit including the H96D, Y114F, L25W and L25W/I10W showed comparable abilities to associate with Gα_q_. Despite the lack of functional regulation of G_q_ signaling by Fhit, stimulation of G_q_-coupled receptors in HEK293 and H1299 cells stably overexpressing Fhit led to reduced cell proliferation, as opposed to an enhanced cell proliferation typically seen with parental cells.

**Conclusions:**

Activated Gα_q_ members interact with Fhit through their α2-β4 region which may result in enhancement of the growth inhibitory effect of Fhit, thus providing a possible avenue for G protein-coupled receptors to modulate tumor suppression.

## Background

The chromosomal localization of *FHIT* (Fragile Histidine Triad) in the common fragile region of the human genome suggests a positive correlation between the loss or inactivation of the *FHIT* gene and carcinogenesis. As predicted for a tumor suppressor, the Fhit protein is absent or markedly reduced in most human cancers [1]. The role of *FHIT* in tumor suppression is perhaps best exemplified by studies performed with *FHIT*-deficient mice. Transgenic mice carrying one or two inactivated Fhit alleles are viable and long-lived, but they show increased rates of spontaneous and carcinogen-induced cancers [2,3]. Encouragingly, the development of carcinogen-induced tumors in these mice can be prevented by administration of Fhit-expressing viral vectors [4]. Moreover, Fhit overexpression enhances the susceptibility of many types of cancer cells to exogenous inducers of apoptosis.

Fhit is one of the HIT (histidine triad) superfamily members, which share an HxHxHxx motif (where x is a hydrophobic residue) for nucleotide binding. Human Fhit can hydrolyze dinucleoside polyphosphates, preferably Ap_3_A (to AMP and ADP). Despite numerous attempts to elucidate the function of Fhit in tumor suppression, the biological action of Fhit remains elusive. Current evidence based on Fhit mutants with impaired substrate binding (L25W and I10W/L25W mutants) or hydrolytic activity (H96D mutant) supports the notion that the formation and stability of the Fhit-Ap_3_A complex is crucial in growth inhibition and apoptosis [5-7]. There is also evidence to suggest that the intracellular concentration of Ap_3_A [8] or its abundance relative to other dinucleoside polyphosphates [9] may be correlated with Fhit-mediated apoptosis. The hypothesis that the Fhit-Ap_3_A complex could be an important signaling molecule is an interesting possibility, but it has yet to be confirmed biochemically.

A number of important cancer-related genes and pathways have recently been linked to Fhit. In colon cancer cell lines, Fhit inhibits cell growth by attenuating the signaling mediated by NFκB [10]. Fhit also inhibits the activity of Akt, a key effector in the phosphatidylinositol 3-OH kinase (PI3K) pathway [11], and serves as a physiological target of the Src tyrosine kinase [12]. Src is a crucial cytoplasmic tyrosine kinase downstream of several growth factor receptors, including those of the EGF receptor family, which are often overexpressed and activated in human breast and ovarian carcinomas. Indeed, activation of EGF receptor family members induces Fhit degradation via the proteasome pathway which purportedly depends on Src-mediated Fhit phosphorylation at Tyr^114^[13]. However, biochemical data suggest that phosphorylation favors the formation and persistence of the Fhit-Ap_3_A complex [14]. Additionally, the mitochondrial Fhit can sensitize cells to apoptosis by binding and stabilizing ferredoxin reductase [15], which is important for the production of reactive oxygen species, and by enhancing mitochondrial Ca^2+^-uptake capacity [16]. These reports help us to better understanding the mechanism of tumor suppression by Fhit, but it remains unclear as to how one can restore Fhit levels in the tumor cells for cancer treatment.

Many signaling pathways operated by growth factors are similarly modulated by the heterotrimeric G proteins, which are critical players in many aspects of cellular function including cell proliferation, differentiation and apoptosis. These signaling pathways include the mitogen-activated protein kinases (MAPKs) [17], PI3K/Akt [18], tyrosine kinases [19], and transcription factors such as STAT3 and NFκB [20,21]. Gα subunits of heterotrimeric G protein are classified into four subfamilies (Gα_s_, Gα_i_, Gα_q_, and Gα_12_) [22]. It is noteworthy that some Gα subunits can directly activate tyrosine kinases such as Bruton's tyrosine kinase (Btk) [19]. Interestingly, Src has also been shown to be activated by members from all four subfamilies of G proteins [23-26] and this may provide a link to regulate Fhit phosphorylation. Constitutively activating mutations of the Gα subunits that lock these signaling molecules in their GTP-bound active state have been found to be associated with several types of tumor [27]. Sustained stimulation of the G_q_ and G_12_ pathways often leads to mitogenesis in various cell types [28]. As a continuing effort to understand the functions of G proteins in cell growth and proliferation, we have explored the notion that G proteins can modulate Fhit. Surprisingly, we discovered that several α subunits of G_q_ family members can associate with Fhit only in their active state.

## Results

### Constitutively active Gα_q_ mutants stimulate Fhit phosphorylation at Tyr^114^ through Src

**Figure 1:**
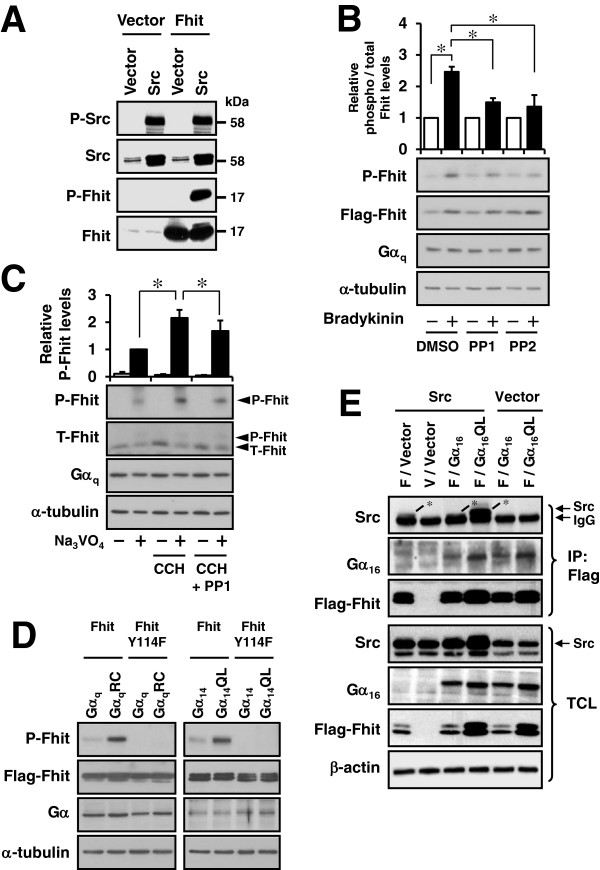
**Activation of Gα**_**q**_**stimulates Fhit Tyr**^**114**^**phosphorylation in a Src-dependent mannar while activated Gα**_**q**_**can associate with Fhit independent of Src.A**, HEK293 cells were co-transfected with either pcDNA3 (Vector) or pcDNA3-Fhit in combination with pcDNA3 or pcDNA3-Src. Phosphorylated Src at Tyr^416^ and phosphorylated Fhit at Tyr^114^ were determined by Western blotting. **B**, HEK293 cells stably expressing type 2 bradykinin receptor were transiently transfected with pFlag-CMV2-Fhit and then seeded onto 6-well plates. One day later, cells were serum starved, pretreated with DMSO, 10 μM PP1 or 25 μM PP2 for 30 min and then treated with or without 100 nM bradykinin for 24 h. The levels of phosphorylated Fhit were quantified relative to the controls without bradykinin treatment (set as 1). * Significantly different from the indicated controls (Dunnett *t* test, P < 0.05). **C**, Serum starved HEK293 cells were pretreated with 10 μM PP1 for 30 min and then treated with or without 100 μM carbachol (CCH) in the absence or presence of 100 μM Na_3_VO_4_ for 24 h. * Carbachol treatment significantly increased the phosphorylation of endogenous Fhit, while inhibition of Src significantly suppressed this effect (Dunnett *t* test, n = 4, P < 0.05). **D**, HEK293 cells were co-transfected with pFlag-CMV2-Fhit or pFlag-CMV2-Fhit Y114F in combination with wild-type or constitutively active mutant of Gα_q_ or Gα_14_. The cDNA amount of Fhit for transfection was adjusted to achieve comparable expression levels. **E**, HEK293 cells were co-transfected with different combinations of pFLAG-CMV2-Fhit (F), pFLAG-CMV2 expression vector (V), Gα_16_, Gα_16_QL, pcDNA3 (Vector) or Src constructs. After 24 h, cells were collected and immunoprecipitated with anti-Flag affinity gel. * The upper bands represent Src while the lower bands were heavy chains of immunoglobulin G. Immunoblots shown represent one of three sets; two other sets yielded similar results.

Src is known to be activated by Gα_q_ subunits [20,25] and thus it is conceivable that stimulation of G_q_-coupled receptors may lead to Fhit phosphorylation. To facilitate the detection of Fhit phosphorylation, we raised an anti-phospho-Fhit Tyr^114^ antiserum which can detect Src-induced Fhit Tyr^114^ phosphorylation with high sensitivity (Figure 1A); overexpression of Src was sufficient to induce Fhit phosphorylation in transfected HEK293 cells due to the increase in activated Src (P-Src in Figure 1A). We then began the study by examining the ability of the G_q_-coupled type 2 bradykinin receptor (BK_2_R) to stimulate Fhit phosphorylation by using a previously characterized HEK293 cell line stably expressing BK_2_R (293/BK_2_R cells) [29]. 293/BK_2_R cells transiently expressing Flag-Fhit were stimulated with or without 100 nM bradykinin for various durations and then assayed for Fhit phosphorylation. Bradykinin-induced Fhit phosphorylation was hardly detected at short treatment times (data not shown) but was reproducibly observed albeit weakly with cells treated for 24 h (~2.5-fold of basal; Figure 1B, DMSO control). As shown in Figure 1B, bradykinin-induced Fhit Tyr^114^ phosphorylation was significantly suppressed by pretreatment of the cells with Src inhibitors (10 μM PP1 or 25 μM PP2). As HEK293 cells endogenously express the G_q_-coupled muscarinic M_3_ receptor [30], we examined whether receptor activation can induce Src-mediated Tyr^114^ phosphorylation of endogenous Fhit. In contrast to 293/BK_2_R cells overexpressing Flag-Fhit, we could not detect carbachol-induced phosphorylation of endogenous Fhit in native HEK293 cells unless the cells were treated with 100 μM Na_3_VO_4_, a tyrosine phosphatase inhibitor (Figure 1C); this suggests that phosphorylated Fhit may undergo dephosphorylation and thereby making its detection extremely difficult when the level of phospho-Fhit is limiting. Nevertheless, the carbachol-induced phosphorylation of endogenous Fhit was sensitive to Src inhibition by PP1 (Figure 1C). In order to confirm that G_q_ signals can lead to Fhit phosphorylation, we made use of constitutively active mutants of Gα_q_ subunits as well as Fhit Y114F, a previously characterized non-phosphorable mutant [12,13]. The constitutively active Gα mutants harbor a point mutation at a conserved arginine or glutamine (e.g., Gα_q_R183C or Gα_q_Q209L) which abolishes the GTPase activity of the Gα subunit and maintains them in the GTP-bound active state. Transient co-expression of constitutively active Gα_q_ mutants with Fhit should lead to increased phosphorylation of wild-type Fhit but not Fhit Y114F. Interestingly, co-expression of constitutively active mutants of Gα_q_ or Gα_14_ (another member of the Gα_q_ family) with Fhit resulted in increased levels of the latter (Additional file 1), a phenomenon similar to that seen with bradykinin-treated 293/BK_2_R cells (*cf* lanes 1 and 2 of the Flag-Fhit immunoblot in Figure 1B). After adjusting the expression level of Fhit between the various transfectants, Fhit phosphorylation was clearly detected in cells co-expressing the constitutively active Gα_q_RC or Gα_14_QL (Figure 1D). Transfectants co-expressing the wild-type Gα subunits exhibited little or no Fhit phosphorylation while no phospho-Fhit could be detected in cells co-expressing Fhit Y114F (Figure 1D).

As tyrosine kinases such as Btk can be directly activated by Gα_q_[19], we examined whether Src can form complexes with Fhit and/or Gα_q_. Because activated Gα_16_ (*GNA15*, another member of Gα_q_ subfamily with 85% sequence identity to its mouse isoform Gα_15_[31]) has previously been shown to stimulate Src phosphorylation at Tyr^416^[21], we transfected HEK293 cell with different combinations of Flag-Fhit, Src, Gα_16_ and Gα_16_QL and then subjected the cell lysates to co-immonuprecipitation assays using an anti-Flag affinity gel (Figure 1E). Both Src and Gα_16_QL were detected in the immunoprecipitates of Flag-Fhit when all three proteins were co-expressed simultaneously (Figure 1E, lane 4); note that the Src-specific band (marked by an asterisk) ran just above a non-specific IgG band. Control experiments omitting either Src or Gα_16_QL demonstrated that both proteins were able to interact with Flag-Fhit independently or endogenous levels of interacting proteins (including Src and Gα_q_ subunits) were not limiting (*cf* lanes 1 and 6 in Figure 1E). Compared to Gα_16_QL, wild-type Gα_16_ exhibited a much weaker ability to associate with Flag-Fhit (*cf* lanes 3 and 5 versus 4 and 6 in Figure 1E). Yet again, co-expression of Gα_16_QL, but not wild-type Gα_16_ or Src, increased the levels of Fhit in the transfectants (Figure 1E, lanes 4 and 6). Taken together, these results suggest that Fhit may associate with Gα subunits in a GTP-bound state-dependent and Src-independent manner.

### Several Gα_q_ members interact with Fhit in an activity-dependent manner

**Figure 2:**
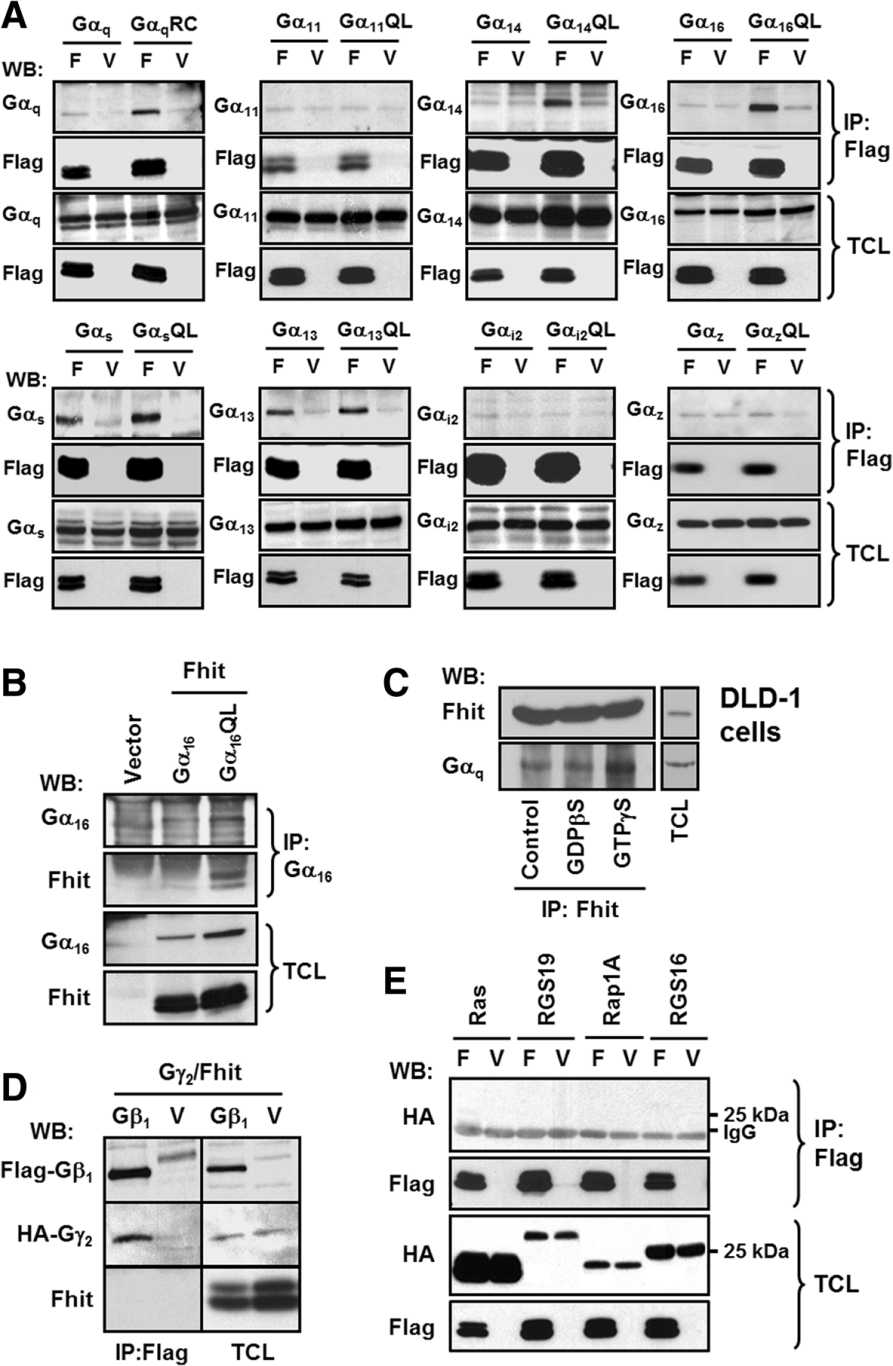
**Fhit interacts with activated Gα**_**q**_**subunits but not with Gβ, small GTPases or RGS proteins.A**, HEK293 cells were co-transfected with either pFlag-CMV2-Fhit (F) or pFlag-CMV2 vector (V) and in combination with individual construct encoding wild-type or the constitutively active mutant (RC for G_q_, QL for the others) of different Gα proteins: G_q_, G_11_, G_14_, G_16_, G_s_, G_i2_ and G_13_. After 24 h overexpression, cell lysates were prepared and subjected to immunoprecipitation (IP) with anti-Flag agarose affinity gel. Total cell lysates (TCL) and the immunoprecipitates were analyzed by Western blotting (WB). **B**, HEK293 cells were transfected with pcDNA3 (Vector) or pFlag-CMV2-Fhit together with Gα_16_ or Gα_16_QL. Transfectants were subjected to IP with anti-Gα_16_ antiserum and protein G agarose. **C**, DLD-1 colon carcinoma cell lysates were incubated without or with GDPβS or GTPγS for 30 min at 4°C and then subjected to IP with anti-Fhit antiserum and protein A agarose. **D**, HEK293 cells were co-transfected with either Flag-Gβ_1_ or pFlag-CMV2 vector with HA-Gγ_2_ and pcDNA3-Fhit. Transfectants were immunoprecipitated with anti-Flag agarose affinity gel. **E,** HEK293 cells were co-transfected with either pFlag-CMV2-Fhit or pFlag-CMV2 vector and in combination with individual HA-tagged construct encoding Ras, RGS19, Rap1A, or RGS16. Cell lysates were immunoprecipitated with anti-Flag agarose affinity gel. Data shown represent one of three or more sets of immunoblots; other sets yielded similar results.

The preceding experiments suggest that members of the Gα_q_ subfamily may interact with Fhit upon binding GTP. To assess if this interaction is specific to Gα_q_ subunits, we performed co-immunoprecipitation assays using Flag-Fhit and various Gα subunits. HEK293 cells were co-transfected with Flag-Fhit or Flag-vector in combination with a selected Gα subunit in its wild-type or constitutively active form. The expressions of Flag-Fhit and Gα subunits between different groups were adjusted to comparable levels prior to co-immunoprecipitation with an anti-Flag affinity gel or anti-Gα antiserum. Constitutively active mutants of Gα_q_, Gα_14_, and Gα_16_, but not their wild-type counterparts, formed complexes with Flag-Fhit as predicted (Figure 2A). However, despite being a member of the Gα_q_ subfamily, the constitutively active mutant of Gα_11_ failed to interact with Flag-Fhit (Figure 2A). Representative members (Gα_s_, Gα_i2_ and Gα_13_) from each of the remaining Gα subfamilies were also subjected to co-immunoprecipitation assays with Flag-Fhit. As shown in Figure 2A, both wild-type and constitutively active Gα_s_ and Gα_13_ were pulled down by Flag-Fhit, but not by the vector control, suggesting that Gα_s_ and Gα_13_ were capable of forming complexes with Flag-Fhit irrespective of their activation status. Neither wild-type nor constitutively active Gα_i2_ or Gα_z_ was co-immunoprecipitated with Flag-Fhit, indicating that both Gα_i2_ and Gα_z_ behaved like Gα_11_ and could not associate with Fhit. To ascertain that Fhit can truly interact with activated members of Gα_q_, we examined the association between Gα_16_QL and Fhit by reciprocal co-immunoprecipitation using an anti-Gα_16_ antiserum to pull down Fhit from lysates of HEK293 cells expressing wild-type Gα_16_ or Gα_16_QL; Fhit was indeed co-immunoprecipitated along with Gα_16_QL, but not with wild-type Gα_16_ (Figure 2B).

To further confirm their interaction in a native system, we screened for cell lines that endogenously express Fhit at a detectable level. Out of eight cell lines examined, DLD-1 colon carcinoma cells have relatively high levels of endogenous Fhit (data not shown) and they were used to examine the interaction between endogenous Fhit and Gα_q_. Cell lysates were incubated with non-hydrolysable GDPβS or GTPγS (100 μM each at 4°C for 30 min) to shift the endogenous G proteins to the basal or activated state, respectively. Cell lysates were subsequently subjected to co-immunoprecipitation with anti-Fhit antiserum and protein A sepharose. As compared to the controls, more Gα_q_ was detected in the Fhit immunoprecipitate following GTPγS treatment (Figure 2C). This result suggests that activated Gα_q_ subunits can interact with Fhit in a native cellular environment.

Since other signaling components along the G protein pathway may also be involved in the Fhit/Gα_q_ interaction, possible association of Fhit with Gβγ, regulators of G protein signaling (RGS proteins), and monomeric GTPases were examined by co-immunoprecipitation assays. Many effectors such as adenylyl cyclase and phospholipase Cβ (PLCβ) can be simultaneously regulated by Gα and Gβγ subunits. It is thus worth investigating whether Fhit can also associate with Gβ_1_γ_2_, a Gβγ complex which is known to bind various effectors including tyrosine kinases [32]. We co-expressed Flag-tagged Gβ_1_ and HA-tagged Gγ_2_ with untagged Fhit. The Flag-Gβ_1_ subunit was clearly capable of forming a complex with HA-Gγ_2_, yet it was unable to co-immunoprecipitate Fhit (Figure 2D). As shown in Figure 2E, both RGS19 (also known as Gα-interacting protein, GAIP) and RGS16 did not co-immunoprecipitate with Flag-Fhit. RGS4, RGS10, and RGS20 also failed to interact with Fhit (data not shown). It should be noted that, under identical experimental conditions, RGS19 and Ras can interact efficiently with their known partners [33,34]. Monomeric small GTPases contain the same core domains for GTP-binding as the heterotrimeric Gα subunits. Hence, the ability of Flag-Fhit to form a complex with selected small GTPases was examined. Neither Ras nor Rap1A, which belong to the Ras family of the small GTPase superfamily, could be co-immunoprecipitated by Flag-Fhit (Figure 2E), suggesting that small GTPases cannot form complexes with Fhit protein. These observations further support the notion that Gα_q_/Fhit interactions are specific and not shared by other signaling components along the G protein pathway.

### Activated Gα_16_ interacts with Fhit directly through its α2-β4 region

**Figure 3:**
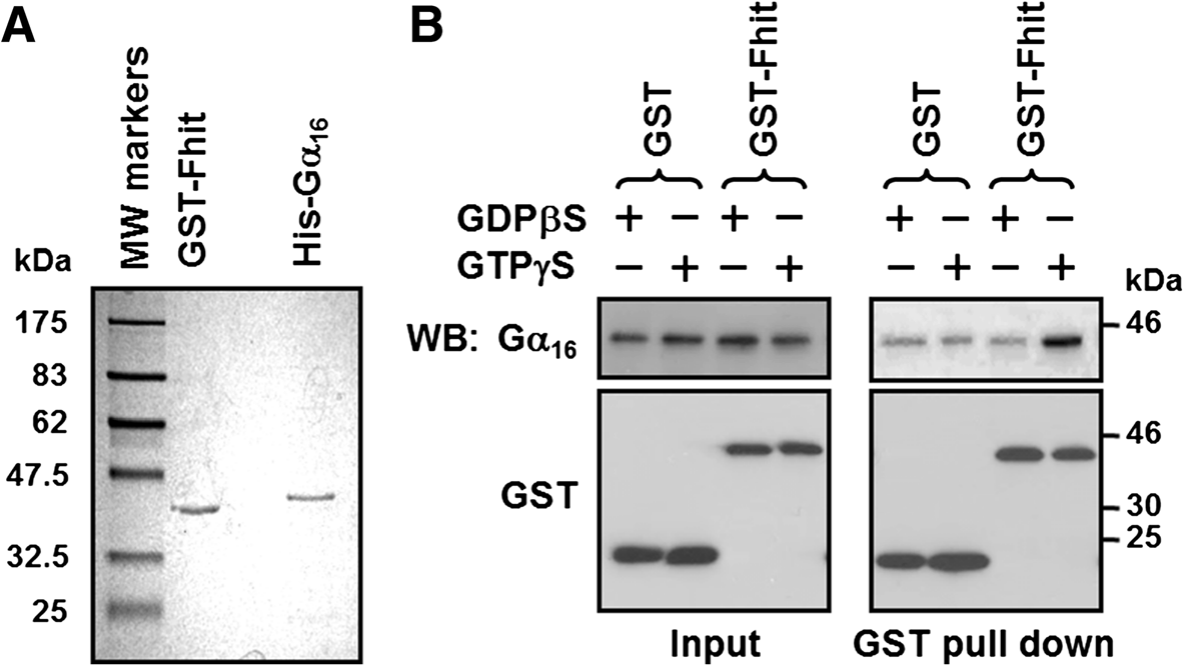
**Fhit interacts directly with activated Gα**_**16**_**.A**, Purified GST-Fhit and His-Gα_16_ were analyzed by Coomassie blue staining. **B**, Equal amounts (2 μg each) of purified GST or GST-Fhit was incubated with either GDPβS-bound or GTPγS-bound His-Gα_16_ at 4°C for 30 min. The mixture was then subjected to GST pull-down assay with glutathione Sepharose beads. The input and GST pull down samples were analyzed by Western blot.

To investigate whether Fhit is able to directly interact with activated Gα_q_ members, we performed pull-down assays using purified GST, GST-tagged Fhit (GST-Fhit) and His-tagged Gα_16_ (His-Gα_16_). The purity of both GST-Fhit and His-Gα_16_ proteins was estimated to be greater than 90% by Coomassie blue staining (Figure 3A). Equal amounts of recombinant His-Gα_16_ and GST-Fhit (or GST) were incubated at 4°C for 30 min in the presence of 100 μM GDPβS or GTPγS in order to stabilize His-Gα_16_ in the inactive or active conformation. Although a small amount of His-Gα_16_ appeared to be non-specifically associated with the glutathione sepharose (Figure 3B, lanes 1 and 2 of right panel), GTPγS-His-Gα_16_ was clearly pulled down by GST-Fhit (Figure 3B, lane 4 of right panel). In contrast, GDPβS-His-Gα_16_ failed to associate with GST-Fhit. Collectively, these results suggest that Fhit can selectively associate with activated Gα_q_ members except Gα_11_, and both purified Gα_16_ and endogenous Gα_q_ can interact with Fhit in their active states. Such activation state-dependent interactions are reminiscent of Gα/effector regulations.

**Figure 4:**
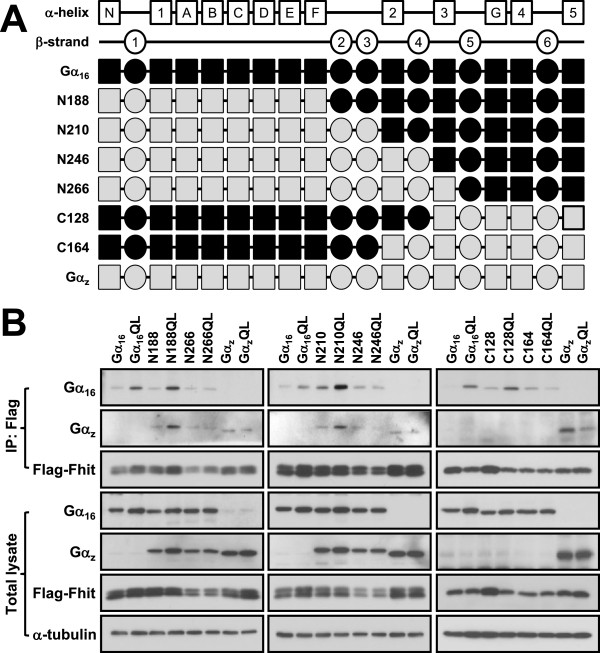
**The α2-β4 region is important for Gα**_**16**_**to interact with Fhit.A**, Schematic representation of the N188, N210, N246, N266, C128 and C164 chimeras. Predicted secondary structures are illustrated as boxes (α helices) or ovals (β strands) above the chimeras. Sequences from human Gα_z_ are shaded in grey while those from human Gα_16_ are in black. **B**, HEK293 cells were transiently co-transfected with Flag tagged Fhit and the wild-type or constitutively active mutants of Gα_16_, Gα_z_, N188, N210, N246, N266, C128 or C164. Cell lysates were immunoprecipitated with anti-Flag agarose affinity gel (upper panels). Expression levels of Gα_16_, Gα_z_, Flag-Fhit and α-tubulin in the total cell lysate were detected by western blotting (lower panels). Data shown represent one of three or more sets of immunoblots; other sets yielded similar results.

In order to understand the molecular basis of the interaction between Gα_q_ and Fhit, we mapped the Fhit-interacting regions on Gα_16_ by using a series of chimeras in which discrete regions of Gα_16_ were swapped with Gα_z_ (a member of Gα_i_ subfamily). These chimeras have been previously used to successfully determine the receptor and effector interacting domains of Gα_16_ and Gα_z_[35,36]. Gα_16/z_ chimeras were preferred because of the lack of endogenous expression of either Gα_16_ or Gα_z_ in HEK293 cells. The differential ability of Gα_16_QL and Gα_z_QL to interact with Fhit (Figure 2A) permits identification of Fhit-interacting regions on Gα_16_ through gain of function analyses. Since the effector interacting domain is likely to reside in the carboxyl half of the Gα subunit [36,37], we have selected chimeras composed of Gα_z_ backbones with their C-terminal regions increasingly replaced by Gα_16_ sequences all the way up to the β2 domain (Figure 4A); mirror images of selected chimeras were also included. Among the various chimeras examined, constitutively active N188QL and N210QL (N-terminal 188 or 210 amino acids from Gα_z_, respectively) were more efficiently pulled down by the anti-Flag affinity gel than their corresponding wild-types; both chimeras were as effective as, if not better than, Gα_16_QL (Figure 4B). Constitutively active C128QL (C-terminal 128 amino acids from Gα_z_) also showed higher affinity with Fhit than its wild-type (Figure 4B). In contrast, N246QL, N266QL and C164QL failed to associate with Flag-Fhit and behaved like the negative control Gα_z_QL (Figure 4B). These results demonstrate that the residues between 210 and 246 of Gα_16_, which represent the regions from α2 to β4, are required for interaction with Fhit. Based on the structures of active Gα_q_ in the complex with p63RhoGEF and RhoA [PDB: 2RGN_A] as well as inactive Gα_q_ with Gβγ complex [PDB: 3AH8], molecular modeling of Gα_16_ predicted that the α2-β4 domain interacts with Gβγ in the inactive state but becomes exposed to the outer surface in the active state (Additional file 2).

We have also attempted to determine the Gα_q_-interacting region on Fhit by constructing a series of Fhit truncation mutants with deletions at either the C- or N-terminus (Additional file 3). However, deletion at either terminus apparently impaired the stability of these mutants because their expressions were hardly detectable unless the transfected cells were treated with the proteasome inhibitor MG132 (Additional file 3). The inadequate expression of these truncation mutants precluded co-immunoprecipitation assays. Nevertheless, expressions of two mutants were enhanced upon co-expression of Gα_q_QL, but not Gα_q_ (Additional file 3). This suggests that interaction with activated Gα_q_ may stabilize Fhit.

### Formation of the Gα_q_/Fhit complex is independent of Fhit’s ability to bind Ap_3_A or be phosphorylated at Tyr^114^

**Figure 5:**
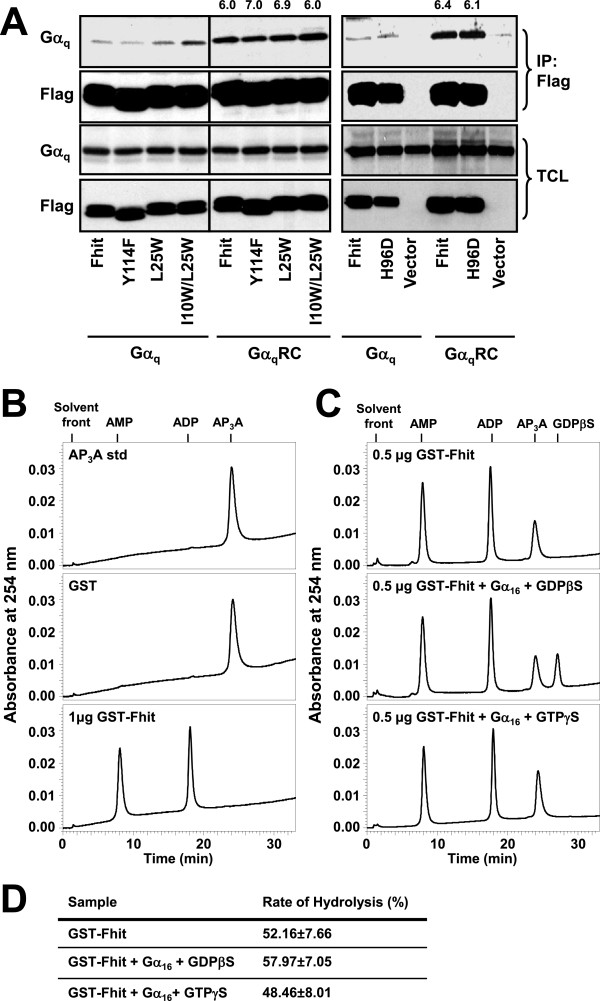
**Interaction with Gα**_**q**_**is not dependent on the ability of Fhit to bind or hydrolyze Ap**_**3**_**A.A**, HEK293 cells were co-transfected with either one of pFlag-CMV2 constructs encoding wild-type Fhit, Fhit-Y114F, Fhit-L25W, Fhit-I10W/L25W, Fhit-H96D or pFlag-CMV2 vector and in combination with wild-type Gα_q_ or constitutively active Gα_q_ mutant (Gα_q_RC). Cell lysates were immunoprecipitated with anti-Flag affinity gel. Numerical values shown above the blot represent the relative intensities of Gα_q_RC being co-immunoprecipitated as compared to their corresponding wild-type Gα_q_. The band intensity of wild-type Gα_q_ pulled down by Flag-Fhit was set as 1.0. Data shown represent one of three or more sets of immunoblots; other sets yielded similar results. **B**, Ap_3_A (100 μM) was incubated in the absence (top) or presence of 1 μg GST protein (middle) or 1 μg GST-Fhit protein (bottom) at 37°C for 10 min and then subjected to HPLC analysis. The elution profiles were compared with HPLC elution profiles of nucleotide standards (data not shown) including Ap_3_A, AMP, ADP, GTPγS and GDPβS; their relative retention times are marked above the reaction profile. **C**, Reaction profiles of Ap_3_A hydrolysis by 0.5 μg GST-Fhit in the absence (top) or presence of 0.5 μg His-Gα_16_ which had been pre-incubated (30°C for 30 min) with 100 μM GDPβS (middle) or 100 μM GTPγS (bottom). The amount of GST-Fhit was reduced to 0.5 μg in order to facilitate the detection of possible stimulatory effect of activated His-Gα_16_. GDPβS was detected as an extra peak (middle) with a retention time of 28.5 min. **D**, Rate of Ap_3_A hydrolysis was analyzed from the profiles shown in **B** and **C**; rate of hydrolysis (%) was expressed as a percentage of Ap_3_A hydrolyzed during the reaction, i.e. percentage difference between areas under the peaks of Ap_3_A before and after the hydrolysis reaction.

In an attempt to unveil the biological function of the Gα_q_/Fhit interaction, we asked if such association is affected by Fhit phosphorylation at Tyr^114^ or Fhit’s ability to bind Ap_3_A. Previous studies have shown that Fhit undergoes degradation upon phosphorylation by Src kinase at Tyr^114^[13] and activated Gα_q_ can stimulate tyrosine kinases [25]. Many signaling molecules regulate their binding to protein partners through tyrosine phosphorylation. To test if this holds true for Fhit, we employed the Fhit Y114F mutant in co-immunoprecipitation assays. Since Flag-Fhit Y114F appeared to interact with constitutively active Gα_q_RC to an extent similar to Flag-Fhit (Figure 5A), it suggests that phosphorylation of Fhit Tyr^114^ is not a prerequisite for the formation of Gα_q_/Fhit complexes.

Ap_3_A is the substrate of Fhit, and binding of Ap_3_A to Fhit can affect the conformation of Fhit and hence its ability to associate with other proteins. I10W/L25W and L25W are Fhit mutants that exhibit 30- and 7-fold increase of *K*_*m*_, respectively [7]. Apparently, these mutants have a lower affinity to associate Ap_3_A although they can still hydrolyze Ap_3_A. On the other hand, H96D, the Ap_3_A hydrolytic dead mutant of Fhit does not hydrolyze Ap_3_A and stabilizes the Ap_3_A/Fhit conformation [6]. Therefore, the associations between Gα_q_ and these mutants were assessed. As shown in Figure 5A, all three mutants effectively co-immunoprecipitated Gα_q_RC but not wild-type Gα_q_; their interactions with Gα_q_RC were essentially similar to that observed with Flag-Fhit. Hence, the binding of Ap_3_A to Fhit has little or no effect on the formation of Gα_q_/Fhit complexes.

Since many activated Gα subunits can regulate the enzymatic activity of their effectors, constitutively active Gα_q_ may modulate the hydrolase activity of Fhit. To test this possibility, we used purified GST-Fhit and His-Gα_16_ proteins. The hydrolysis of Ap_3_A to AMP and ADP was monitored by HPLC as described previously [38]. Upon incubation with 1 μg GST-Fhit at 37°C for 10 min, 100 μM Ap_3_A was completely hydrolyzed to AMP and ADP (Figure 5B). No hydrolysis was detected when Ap_3_A was incubated with GST alone or with heat denatured GST-Fhit (Figure 5B). We then optimized the assay in order to cater for the detection of possible stimulatory effect on the hydrolase activity of Fhit. Upon reducing the amount of GST-Fhit in the reaction to 0.5 μg, approximately half of the Ap_3_A was hydrolyzed to AMP and ADP (Figure 5C). To mimic the constitutively active Gα_16_QL, the recombinant His-Gα_16_ protein was loaded with 100 μM GTPγS. His-Gα_16_ protein loaded with GDPβS was used as a negative control. As shown in Figure 5C, the presence of GTPγS-bound or GDPβS-bound His-Gα_16_ did not affect the ability of GST-Fhit to hydrolyze Ap_3_A. The extent of Ap_3_A hydrolysis by GST-Fhit was essentially identical under all three conditions (Figure 5D). Neither guanine nucleotides interfered with the detection of the substrate or product; GDPβS was eluted after Ap_3_A while GTPγS could not be detected under our experimental conditions. These results suggest that activated Gα_16_ does not regulate the hydrolase activity of Fhit. However, it remains possible that activated Gα_16_ can indirectly modulate the enzymatic activity of Fhit in a cellular environment.

### Fhit does not alter the signaling function of Gα_q_

**Figure 6:**
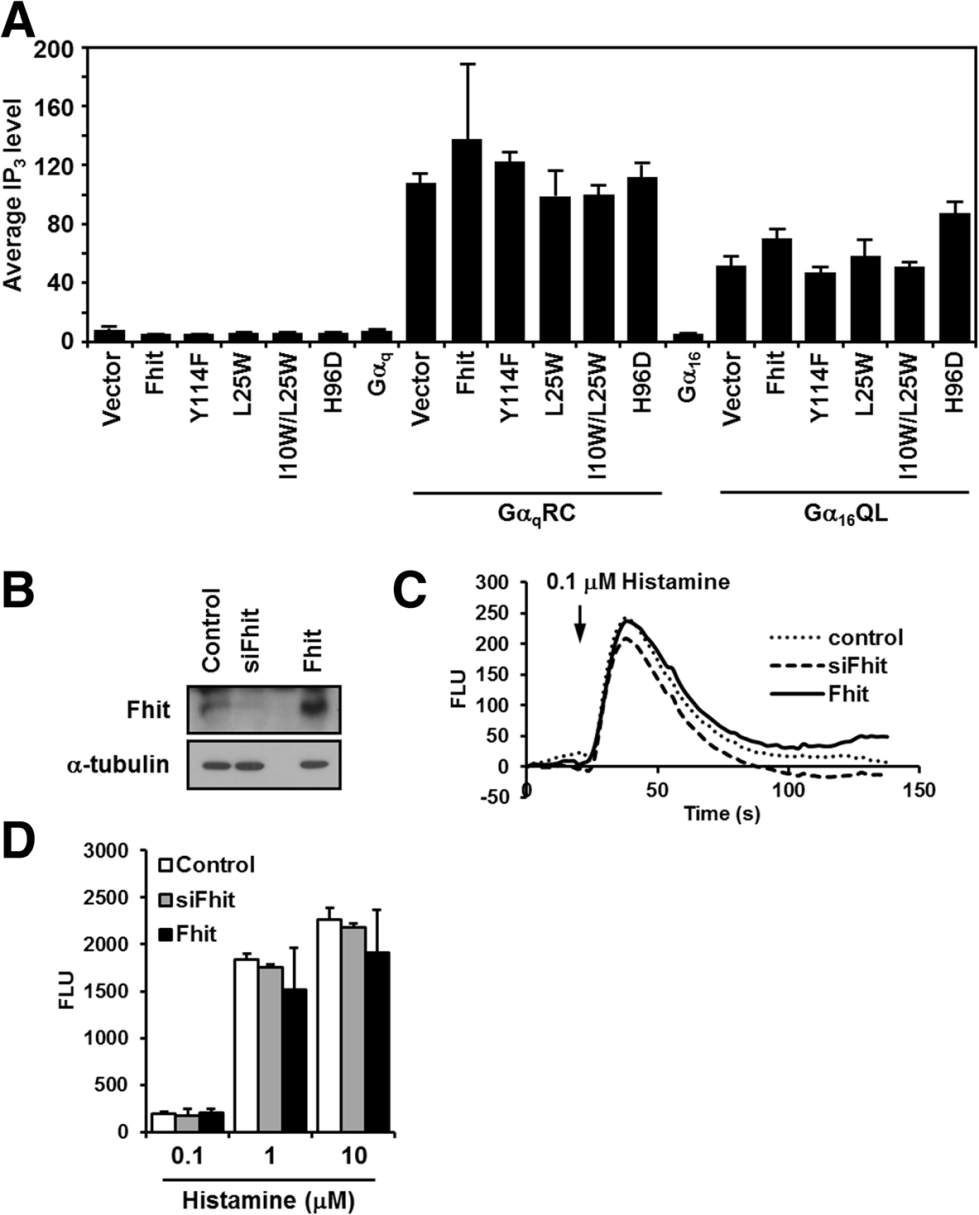
**Fhit does not affect G**_**q**_**-mediated PLCβ activation.A**, HEK293 cells were co-transfected with wild-type or the constitutively active mutant of Gα_16_ or Gα_q_ in combination with pcDNA3 (Vector), Fhit and different Fhit mutants in the pcDNA3 vector: Fhit-Y114F (Y114F), Fhit-L25W (L25W), Fhit-I10W/L25W (I10W/L25W) and Fhit-H96D (H96D). Transfectants were then labeled and lysed for determining the IP_3_ in cell lysates as described in Methods. PLC activity was calculated as the amount of IP_3_ formed divided by the corresponding level of total inositol. **B**, HeLa cells were transfected with control siRNA, Fhit siRNA or Fhit cDNA. One day later, expression levels of Fhit were detected by Western blot. **C**, Hela cells in **B** were seeded into black-walled 96-well plates and the Ca^2+^ responses of these cells with histamine treatment (0.1, 1, or 10 μM) were detected by the FLIPR device. Here shows the fluorescence signals of 0.1 μM histamine-induced Ca^2+^ response (FLU) in the control (dotted), Fhit-knocked down (dashed) or Fhit-overexpressing (solid) HeLa cells. **D**, The maximal fluorescence signals of the Ca^2+^ responses (FLU) induced by 0.1, 1, or 10 μM histamine in the control, Fhit-knocked down and Fhit-overexpressing HeLa cells were illustrated as white, gray and black, respectively.

As members of the Gα_q_ family are known to regulate mitogenic pathways [39], Fhit may exert its tumor suppressive effect by altering the functions of these Gα subunits. To test this postulation, we determined the effect of Fhit on the ability of Gα_q_ and Gα_16_ to regulate a panel of known effectors. We first examined the ability of Gα_q_RC and Gα_16_QL to stimulate PLCβ in the absence or presence of Fhit overexpression. HEK293 cells were co-transfected with various combinations of Fhit, Fhit mutants, and wild-type or constitutively active mutants of Gα_q_ and Gα_16_. As predicted, both Gα_q_RC and Gα_16_QL were capable of stimulating the endogenous PLCβ and inducing the formation of IP_3_ (Figure 6A). Co-expression of Fhit or its mutants neither stimulated nor inhibited the ability of Gα_q_RC and Gα_16_QL to activate PLCβ (Figure 6A). We have also examined whether Fhit affects the ability of endogenous G_q_-coupled histamine receptors to stimulate PLCβ activity in HeLa cells. As there are conflicting results on the Fhit expression level in HeLa cells [16,40-43], we have confirmed that the HeLa cells used in our study do express endogenous Fhit to a level slightly higher than that seen with HEK293 cells (Additional file 4), which are known to express Fhit [8]. Variations in the reported Fhit levels in HeLa cells might be attributed to differences in the gene expression profiles of sublines. After knocking down of Fhit by siRNA or overexpression of Fhit in HeLa cells (Figure 6B), intracellular Ca^2+^ mobilization was measured by a FLIPR device with 0.1, 1 or 10 μM histamine as agonist. Figure 6C showed typical Ca^2+^ signals induced by 0.1 μM histamine. There was no significant difference among the maximal Ca^2+^ responses induced by different concentrations of histamine in control, Fhit-deficient or Fhit-overexpressing cells (Figure 6D). These observations suggest that Fhit does not affect the Gα_q/16_/PLCβ pathway.

**Figure 7:**
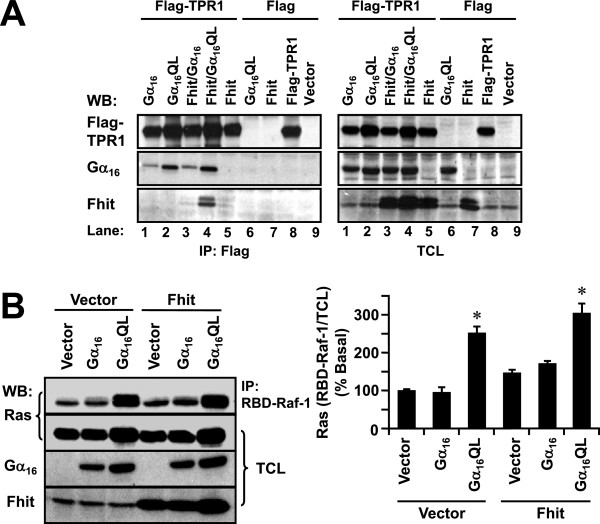
**Fhit does not affect G**_**q**_**-mediated TPR1 interaction or Ras activation.A**, HEK293 cells were co-transfected with different combinations of pcDNA3 (Vector), Fhit, Gα_16_, Gα_16_QL, Flag-TPR1 and Flag tag constructs. One day later, cells were immunoprecipitated with anti-Flag agarose affinity gel and probed for the presence of Fhit, Gα_16_, and Flag-TPR1 in the immunoprecipitates using specific antibodies as indicated. **B**, HEK293 cells were co-transfected with either pcDNA3 (Vector) or Fhit in combination with pcDNA3, Gα_16_ or Gα_16_QL, and subjected to Ras activation assay. The results shown on the bar chart represent relative band intensities of Ras from RBD-Raf-1 immunoprecipitation normalized against their corresponding amounts of total Ras (RBD-Raf-1/TCL). The value of vector-transfected basal control was set as 100%. * Transfection of Gα_16_QL significantly induced Ras activity as compared to vector control (Dunnett’s *t* test, P < 0.05). Immunoblots shown represent one of three sets; two other sets yielded similar results.

Apart from PLCβ, Gα_q_ subunits are known to interact with TPR1 which associates with activated Ras [34,44]. This raises the question whether Fhit could interfere with Gα_16_QL/TPR1/Ras signaling. If Fhit and TPR1 compete for the same region on Gα_16_QL, Fhit will displace and prevent TPR1 from binding to Gα_16_QL. In co-immunoprecipitation assays, the ability of Flag-TPR1 to pull down Gα_16_QL was not affected by the co-expression of untagged-Fhit (Figure 7A, lane 2 versus lane 4), suggesting that TPR1 and Fhit do not compete for the same region on Gα_16_QL. Interestingly, Fhit was clearly present in the immunoprecipitates of lysates prepared from Flag-TPR1/Fhit/Gα_16_QL transfectants (lane 4), whereas it was weakly detected from those of Flag-TPR1/Fhit (lane 5) and Flag-TPR1/Fhit/Gα_16_ (lane 3) transfectants. This might occur if Gα_16_QL could simultaneously bind to both Fhit and Flag-TPR1, thus forming a TPR1/Gα_16_QL/Fhit complex that can be immunoprecipitated by the anti-Flag antibody. The presence of such a complex implies that Fhit may be involved in regulating Gα_16_QL-mediated Ras activation. Ras activation assay was employed to investigate the effect of Fhit on Gα_16_QL-induced Ras activity. In agreement with a previous report [44], Gα_16_QL significantly induced Ras activation as compared to the vector control and wild-type Gα_16_ (Figure 7B). However, there was no significant elevation or attenuation of Ras activity when cells were co-transfected with Fhit (Figure 7B).

**Figure 8:**
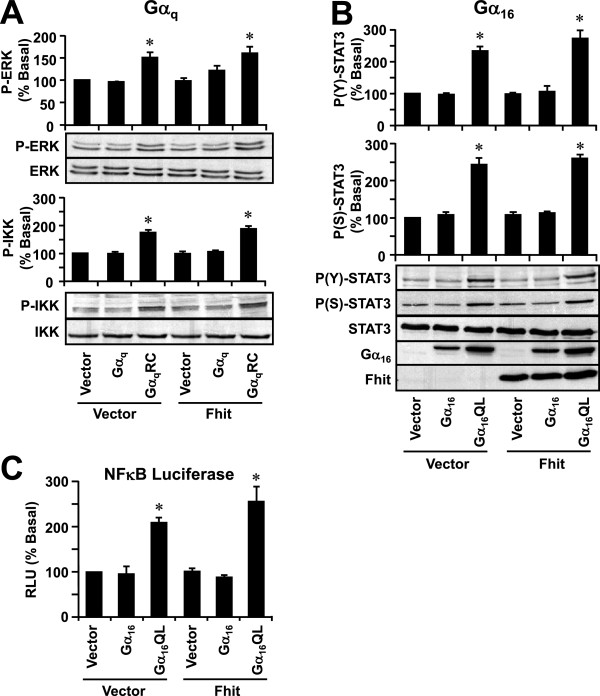
**Overexpression of Fhit does not affect constitutively active Gα**_**q**_**or Gα**_**16**_**-induced phosphorylation of ERK, IKK, or STAT3, or the activity of NFκB.A**, HEK293 cells were co-transfected with either pcDNA3 (Vector) or Fhit in combination with pcDNA3, Gα_q_ or Gα_q_RC. Cell lysates were prepared and immunoblotted with anti-phospho-ERK (P-ERK), anti-ERK (ERK), anti-phospho-IKK (P-IKK), or anti-IKK (IKK). **B**, HEK293 cells were transfected as in *A* except the Gα_q_ constructs were replaced with those corresponding to Gα_16_. Cell lysates were probed with anti-phospho-Tyr^705^-STAT3 (P(Y)-STAT3), anti-phospho-Ser^727^-STAT3 (P(S)-STAT3) or anti-STAT3 (STAT3) antiserum. The vector transfection control was set as 100% control. * The level of ERK, IKK and STAT3 phosphorylation was significantly higher than the vector control (Dunnett’s *t* test, p < 0.05). Immunoblots shown represent one of at least three sets; all other sets yielded similar results. **C**, HEK293 cells stably expressing the NFκB luciferase reporter gene, pNFκB-TA-luc, were co-transfected with either pcDNA3 (Vector) or Fhit in combination with pcDNA3, Gα_16_ or Gα_16_QL. One day later, transfectants were subjected to luciferase assay. * Expression of Gα_16_QL significantly induced NFκB stimulation as compared to pcDNA3 control (Dunnett’s *t* test, P < 0.05).

In addition to PLCβ and Ras signaling, other cytoplasmic signaling molecules known to be regulated by Gα_q_ and Gα_16_ were examined in the presence or absence of Fhit expression. Phosphorylation states of various signaling molecules including ERK, STAT3 and IKK were examined using phospho-specific antibodies. Gα_q_RC significantly stimulated the phosphorylations of ERK and IKK and such responses were unaffected by the presence of Fhit (Figure 8A). Similar results were obtained with Gα_16_QL (data not shown). Likewise, Gα_16_QL significantly stimulated STAT3 phosphorylation at both Tyr^705^ and Ser^727^ and these responses were not affected by the co-expression of Fhit (Figure 8B); similar results were obtained with Gα_q_RC (data not shown).

Since phosphorylation of IKK results in activation of NFκB transcription, Gα_16_QL-stimulated NFκB transcriptional activity was also evaluated. As shown in Figure 8C, Gα_16_QL significantly induced NFκB luciferase activity as compared to pcDNA3 and Gα_16_ control. Consistent with the phosphorylation profiles of IKK, expression of Fhit did not affect the Gα_16_QL-stimulated NFκB transcriptional activity.

### G_q_ activation enhanced the growth inhibitory effect of Fhit

**Figure 9:**
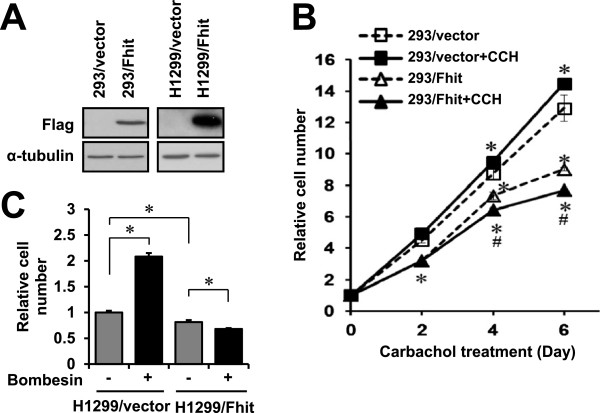
**Stimulation of G**_**q**_**-coupled receptors inhibits cell growth in Fhit-expressing cells.A**, 293/Fhit and 293/vector cells, or H1299/Fhit and H1299/vector cells, which stably expressed Flag-tagged Fhit and the Flag tag alone (vector), respectively, were established as described in Methods. The expressions of Fhit were confirmed by Western blot. **B**, 293/vector or 293/Fhit cells were seeded into 96-well plates and 24 h later, the cells were treated with or without 100 μM carbachol in the growth medium (Day 0). MTT assay were performed on Day 0, 2, 4 and 6 to examine the relative viable cell number. Absorbance values on Day 0 were set as 1 for the respective group of cells. * Significantly different from that of the 293/vector cells without carbachol treatment on the same day; # carbachol treatment significantly inhibited the growth of 293/Fhit cells; Dunnett’s *t* test, p < 0.05, n = 8. **C**, H1299/vector or H1299/Fhit cells were seeded into 96-well plates and 24 h later, the cells were treated with or without 100 nM bombesin (Day 0). MTT assays were performed on Day 0 and 4. The values of MTT assay on Day 0 were set as 1 for the respective group of cells. * Bombesin significantly increased the growth of H1299/vector cells while it significantly inhibited the growth of H1299/Fhit cells; Dunnett’s *t* test, p < 0.05, n = 8.

As Fhit is a tumor suppressor, we asked whether the growth inhibitory effect of Fhit could be affected upon activation of G_q_-coupled receptors. HEK293 and H1299 cells were chosen for this part of the study because they endogenously express G_q_-coupled muscarinic M_1_ and gastrin-releasing peptide receptors (GRPRs), respectively. We established 293/Fhit cells and H1299/Fhit cells stably expressing Fhit (Figure 9A). Prolonged stimulation of G_q_-coupled receptors is often associated with mitogenesis [28], and thus treatment of 293/vector cells with 100 μM carbachol for 4 days or more significantly stimulated cell growth (Figure 9B). In contrast, carbachol significantly inhibited the growth of 293/Fhit cells (Figure 9B); it should also be noted that 293/Fhit cells exhibited reduced growth rate as compared to the 293/vector cells (Figure 9B). A similar effect was observed in H1299 cells. Bombesin has previously been shown to stimulate the proliferation of non-small lung cancer cells including H1299 cells [45,46]. In the present study, activation of GRPR by 100 nM bombesin for 4 days significantly increased the growth of H1299/vector cells but it suppressed the growth of H1299/Fhit cells (Figure 9C). These data suggest that mitogenic responses elicited by G_q_ activation are re-directed into growth suppressive signals when the level of Fhit is elevated. This switching of functional outcome is consistent with the notion that the tumor suppressive action of Fhit is correlated to its expression level [47].

## Discussion

Receptors coupled to members of the Gα_q_ subfamily mediate a wide range of diverse cellular responses, ranging from cell growth and proliferation to cell differentiation [39]. Established models indicate that the actions of G_q_-linked receptors are mediated by inositol lipid signaling, but growing evidence suggests that these pathways alone cannot account for all of the responses. Instead, the extensive list of diverse cellular events involving Gα_q_-linked signals suggests that Gα_q_ subfamily members have multifaceted roles in signal transduction which are not yet fully appreciated. The present study has demonstrated that activated Gα_q_ subunits can directly interact with Fhit, a tumor suppressor widely implicated in many types of cancer [1]. This is especially interesting in view of the ability of Gα_q_ subunits to modulate cell growth and proliferation through regulating critical signaling pathways [48].

The interaction between Gα subunits and Fhit exhibits a high degree of selectivity as demonstrated by the lack of association of Fhit with Gβγ, monomeric GTPases, and RGS proteins. Among the four subfamilies of Gα subunits, at least three can interact with Fhit. Although Gα_i2_ is often regarded as a representative member of the G_i_ subfamily, its inability to interact with Fhit does not necessarily indicate that the other eight Gα_i_ members cannot be partners of Fhit. Likewise, one cannot exclude the possibility that some specific combinations of Gβγ can interact with Fhit unless all viable permutations have been tested. Since both the wild-type and constitutively active mutants of Gα_s_ and Gα_13_ associate with Fhit equally well, such interactions may not be subjected to dynamic cell signaling regulations. Far more interesting is the activation state-dependent interaction between Gα_q_ subunits and Fhit. Activation of Gα_q_ subunits by agonist-bound receptor is expected to drive the formation of Gα_q_/Fhit complexes. Our data suggest that Fhit can indeed interact with activated Gα_q_ in a native cellular environment (Figure 2C) and it can directly associate with activated Gα_16_*in vitro* (Figure 3B). It is noteworthy that the Gα subunits are attached to the inner leaflet of the plasma membrane through fatty acylation and thus Fhit needs to be present at the plasma membrane in order to interact with Gα subunits productively. Analysis of Fhit protein expression in subcellular fractions of normal rat tissue suggests that it is localized at the plasma membrane and the nucleus [49]. Hence Fhit can be in close proximity to Gα_q_ subunits for efficient interactions.

The inability of Gα_11_ to interact with Fhit is rather surprising. The ubiquitously expressed Gα_11_ exhibits 90% sequence homology to Gα_q_ and is thus more closely related to Gα_q_ than the primarily hematopoietic Gα_14_ and Gα_16_[22], and yet the latter two could interact with Fhit as effectively as Gα_q_. No report has indicated any major difference between Gα_11_ and Gα_q_ both in terms of receptor coupling and effector regulation [39]. The ability of Fhit to distinguish Gα_11_ from Gα_q_ as well as Gα_14_ and Gα_16_ thus represents a unique feature of Fhit, but no immediate clue can be drawn as to why it does not form a complex with Gα_11_.

The use of Gα_16/z_ chimeras has enabled us to identify the α2-β4 region of Gα_16_ as an Fhit-interaction domain (Figure 4). This region has been shown to interact with Gβγ complex in the GDP bound Gα_q_ but it becomes available for effector interaction when Gα_q_ adopts the active GTP-bound conformation (Additional file 2). In different Gα_q_ members, this region associates with various effectors such as p63RhoGEF [50] and PLCβ [51]. The binding of Fhit to the α2-β4 region may thus account for the preference of Fhit for constitutively active Gα_q_ mutants that are dissociated from the Gβγ dimers.

The interaction of Gα with Fhit opens a host of possibilities in terms of their biochemical and cellular consequences. Given the known functions of Gα subunits as signal transducers and that only activated Gα_q/14/16_ can interact with Fhit, perhaps the most logical prediction is that Fhit acts as an effector of Gα. If this hypothesis is correct, then activated Gα subunits may affect the localization, stability, or function of Fhit. However, there is a lack of effect of Gα_16_QL on the Ap_3_A hydrolase activity of Fhit. Because Fhit binds and hydrolyzes Ap_3_A *in vitro*[38], any model of Fhit function should take this into account. The ability of GST-Fhit to hydrolyze Ap_3_A into AMP and ADP was, however, unaffected by either GDPβS- or GTPγS-bound His-Gα_16_. Moreover, Fhit mutants with impaired affinity for Ap_3_A (L25W and I10W/L25W) or a lack of hydrolase activity (H89D) formed complexes with activated Gα_q_ subunits as effectively as wild-type Fhit (Figure 5A). These results suggest that activated Gα_q_ subunits have little effect, if any, on the enzymatic activity of Fhit. However, it should be noted that because the catalytic mechanism of Fhit requires leaving-group exit and water entry at the substrate-exposed surface of the dimeric enzyme, polypeptides that bind to the Fhit-Ap_n_A complex are expected to stabilize the complex and retard turnover [6]. Subtle changes in the *K*_*m*_ and/or *K*_*cat*_ of Ap_3_A hydrolysis by Fhit will require detailed kinetic studies.

Equally disappointing is that the formation of the Gα_q_/Fhit complex was unable to interfere with any of the known signaling pathways triggered by Gα_q_. The canonical effector molecules of activated Gα_q_ subunits are the various isoforms of PLCβ. Despite the fact that PLCβ also binds to the Fhit-interacting α2-β4 region of Gα_q_[51], overexpression of wild-type Fhit or its mutants did not affect Gα_q_RC- or Gα_16_QL-induced PLCβ activity (Figure 6A). Activated Gα_q_ may have a higher affinity and preference for PLCβ, resulting in the almost instantaneous formation of IP_3_ and mobilization of intracellular Ca^2+^ (agonist-induced Ca^2+^ mobilization peaks within 10–15 s; Figure 6C). The co-localization of Gα_q_ and PLCβ in lipid rafts [52] helps to ensure the efficiency of the G_q_/PLCβ pathway. Fhit and other effectors may bind to the activated Gα_q_ when the latter becomes dissociated from PLCβ. In this scenario, Fhit would not be able to compromise PLCβ signaling effectively. However, it should be noted that overexpression of p63RhoGEF can inhibit Gα_16_QL-induced PLCβ activity albeit only partially [53] and the presence of Fhit in lipid rafts remains to be confirmed. Fhit can apparently associate with the Gα_16_QL/TPR1 complex since it is detected in the Gα_16_QL/TPR1 immunoprecipitates but not in the absence of Gα_16_QL (Figure 7A). The possible existence of an Fhit/Gα_16_QL/TPR1 complex suggests that Fhit binds to Gα_16_QL on a region distinct from that of TPR1, and this is in agreement with our mapping of the Fhit-interaction domain by using the Gα_16/z_ chimeras (Figure 4) and the fact that TPR1 interacts with the β3 domain of Gα_16_[36]. The lack of effect of Fhit on Gα_16_QL-induced Ras activation further suggests that co-expression of Fhit would not affect the activities of signaling molecules downstream of Ras. This is indeed true for ERK, STAT3, IKK, and NFκB (Figure 8).

**Figure 10:**
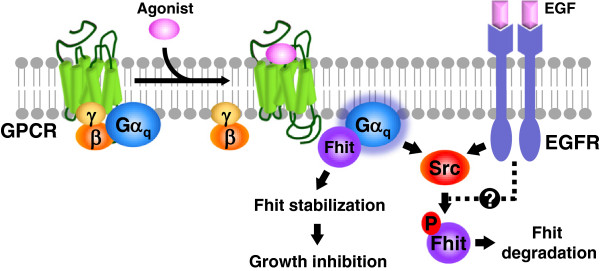
**Distinct regulations of Fhit by G**_**q**_**- and EGF-dependent pathways.** Agonist binding to G_q_-coupled receptor leads to Gα_q_ activation and dissociation with Gβγ complex. Activated Gα_q_ can interact with Fhit and stabilize it, which results in increased Fhit level and consequent enhancement of the growth suppressive effect of Fhit. On the other hand, activation of the EGF receptor stimulates Src-mediated phosphorylation of Fhit at the Tyr^114^ site. The phosphorylated Fhit undergoes degradation which leads to a decrease in the Fhit protein level as well as the tumor suppressive effect of Fhit. Although activated Gα_q_ also stimulates Src-mediated Fhit Tyr^114^ phosphorylation, the overall Fhit protein amount is increased rather than decreased, indicating that either an additional signal is required for the induction of Fhit degradation (which is concomitantly generated by EGF but not by activated Gα_q_; indicated as a dashed line) or activated Gα_q_ can up-regulate Fhit via stabilization.

Although the interaction of activated Gα_q_ and Fhit is independent of the ability of Fhit to become phosphorylated or to bind and hydrolyze Ap_3_A, activation of Gα_q_ could apparently increase Fhit Tyr^114^ phosphorylation through Src (Figure 1B), stabilize Fhit (Figure 1B and Additional file 1, Additional file 3) and enhance the cell growth inhibition effect of Fhit (Figure 9). G_q_ signals often lead to increased cell growth [28], but by forming a complex with Fhit which can stabilize Fhit, activation of Gα_q_ may result in reduced cell growth (Figure 9). Given that activation of EGF receptors triggers the degradation of Fhit [13], and despite the demonstrated ability of activated Gα_q_ to stimulate Fhit phosphorylation (Figure 1B-C), it is rather puzzling to observe that activated Gα_q_ can apparently increase the levels of Fhit (Figure 1B and Additional file 1) and stabilize the truncation mutants of Fhit (Additional file 3). The divergent regulatory outcome of phosphorylated Fhit may be attributed to the differing signaling capacities of EGF- and G_q_-dependent pathways, which could lead to conditional proteasomal degradation of Fhit (Figure 10). An alternative explanation is that Fhit becomes less susceptible to degradation upon binding activated Gα_q_, and this might lead to an elevated level of Fhit (Figure 10). Increased Fhit levels can lead to the suppression of cell proliferation (Figure 9B and C; [4]), while the knock down of Fhit by siRNA increases the viability of DLD-1 cells [10]. If activation of Gα_q_ can elevate the level of Fhit, this might account for the ability of G_q_-coupled receptors to inhibit cell proliferation (Figure 9B and C; [48]). Further investigations are required to elucidate the mechanism by which activated Gα_q_ regulates the level of Fhit. We are currently pursuing the notion that Gα_q_ stimulates the translation of Fhit as we have preliminary data to suggest that the up-regulation of Fhit is blocked by cycloheximide. Since the expression level of Fhit may determine its functional outcome [47], it is tremendously important that quantification of Fhit should be carefully determined in any cellular system to be employed. It should also be noted that Fhit expression can enhance the effects of the p53 tumor suppressor [54] by modulating p53-regulated genes [55]. Hence, the functional relevance of Gα_q_/Fhit interaction should be revisited in experimental systems with different p53 status.

## Conclusions

The present study provides multiple indications that several members of the Gα_q_ family can bind to the tumor suppressor Fhit in their GTP-bound active state. The Fhit-interaction domain on the Gα subunit was identified as the α2-β4 region which would be occluded by the Gβγ dimer in the GDP-bound inactive heterotrimeric G_q_ protein, thus accounting for the preference of Fhit to bind activated forms Gα_q_ subunits. Neither the hydrolase activity of Fhit nor the signaling capacity of activated Gα_q_ was affected by the formation of activated Gα_q_/Fhit complexes. In cells with elevated levels of Fhit, activation of G_q_-coupled receptors led to growth suppression rather than stimulation. Consistent with the tumor suppressive function of Fhit, these observations suggest that the formation of Gα_q_/Fhit complex may modulate cell proliferation.

## Methods

### Reagents

Human cDNAs of various Gα subunits were obtained from Guthrie Research Institute (Sayre, PA). Wild-type Fhit in pCMV-SPORT6 was purchased from Invitrogen (Carlsbad, CA). pRcCMV-Fhit Y114F was a generous gift from Dr. K. Huebner (Comprehensive Cancer Center and Department of Molecular Virology, Immunology, and Medical Genetics, Ohio State University). L25W, I10W/L25W, and H96D mutants of Fhit were kindly provided by Dr. C. Brenner (Department of Genetics and Biochemistry, Dartmouth Medical School). Cell culture reagents, including LipofectAMINE PLUS reagents were purchased from Invitrogen (Carlsbad, CA).Anti-Gα_16_ and anti-Gα_14_ were obtained from Gramsch Laboratories (Schwabhausen, Germany). Anti-Fhit antibody was from Invitrogen (Carlsbad, CA). Anti-G_q/11_ α-subunit antibody was purchased from Calbiochem (San Diego, CA). Anti-α-tubulin antibody, anti-HA antibody, anti-Flag antibody and anti-Flag affinity gel were from Sigma-Aldrich (St. Louis, MO). Antisera against Gα_s_, Gα_i2_ and Gα_13_ were purchased from Santa Cruz Biotechnology (Santa Cruz, CA). Anti-GST antibody was from Abcam (Cambridge, UK). Anti-phospho-Fhit-Tyr^114^ antibody was raised in rabbits against a synthetic peptide corresponding to AA 106–122 of human Fhit containing the phosphorylated tyrosine residue and an additional N-terminally cysteine residue for coupling (C-DFHRNDSI(pY)EELQKHDK). Antibodies were affinity-purified using the immunizing phospho-peptide coupled to SulfoLink® Agarose beads from Thermo Scientific (Rockford, IL) and subsequently cross-absorbed against the non-phosphorylated peptide. Specificity of antibodies was verified by Western blot using cell lysates prepared from HEK293 cells transiently transfected with cDNAs of Fhit or Fhit and Src. Other antibodies were purchased from Cell Signaling Technology (Danvers, MA). GDPβS and GTPγS were from Calbiochem (San Diego, CA). Protein G-agarose and dithiobis[succinimidylpropionate] (DSP) cross-linker were from Pierce Biotechnology (Rockford, IL). ECL kit and Glutathione Sepharose^TM^ 4 Fast Flow beads were from Amersham Biosciences (Piscataway, NJ). Ni-NTA Agarose was obtained from Qiagen (Valencia, CA). Ras activation kit was a product of Upstate-Millipore (Billerica, MA).

### Construction of G protein chimeras and truncation mutants of Fhit

The Gα chimeras (except C128) were constructed as described previously [36] by PCR method using human Gα_16_ and Gα_z_ cDNAs. Briefly, the N-terminal 188, 210, 246 and 266 amino acids or the C-terminal 128 and 164 amino acids of Gα_16_ were swapped to the corresponding regions of Gα_z_ to generate N188, N210, N246, N266, C128 and C164. Primers were designed to cover the overlapping regions of the chimeras, so that 5′ and 3′ fragments can be annealed together to obtain the full length chimeras by PCR. Then the full length PCR products were subcloned into the pcDNA3 vector. All chimeras were confirmed by dideoxynucleotide sequencing. Primer sequence for constructing C128 is 5′- GTG CCT GGA GGA GAA CAA CCA GAC AAG TCG GAT GGC AG-3′.

Flag-tagged Fhit truncation mutants, F131N, F95N, F50C and F27C, were constructed by PCR method using the human Fhit cDNA as a template. The primers were designed based on the secondary structure of Fhit. The outer forward and reverse primers of Fhit are 5′-CGA AGC TTA TGG ACT ACA AAG ACG ATG ACG ACA AGT CGT TCA GAT TTG GCC AAC ATC TC-3′ and 5′- CCT CGA GTC ACT GAA AGT AGA CCC GCA GAG CTG C-3′, respectively. The reverse primers of F131N and F95N are 5′-CCT CGA GTC ATG ATC TCC AAG AGG CAG GAA AGT C-3′ and 5′-CCT CGA GTC AAA CGT GCT TCA CAG TCT GTC CGG C-3′, respectively. The forward primers of F50C and F27C are 5′- CGA AGC TTA TGG ACT ACA AAG ACG ATG ACG ACA AGC TGC GTC CTG ATG AAG TGG CCG-3′ and 5′- CGA AGC TTA TGG ACT ACA AAG ACG ATG ACG ACA AGA ATA GGA AAC CTG TGG TAC CAG GAC-3′, respectively. All truncation mutants were confirmed by dideoxynucleotide sequencing.

### Cell culture and co-immunoprecipitation

HEK293, DLD-1, HeLa and H1299 cells were obtained from the American Type Culture Collection (CRL-1573, Rockville, MD). They were maintained in Eagle’s minimum essential medium (HEK293), RPMI-1640 medium (DLD-1 and H1299) or ATCC-formulated Eagle’s minimum essential medium (HeLa) at 5% CO_2_, 37°C with 10% fetal bovine serum, 50 units/mL penicillin and 50 μg/mL streptomycin.

For co-immunoprecipitation experiments, HEK293 cells were grown to 80% confluency in 100 mm tissue culture plates and then co-transfected with various combinations of cDNAs (3 μg/plate) using 15 μL PLUS and LipofectAMINE reagents in MEM. Serum was replenished 3 h after transfection. Cross-linking was performed one day after transfection; transfected HEK293 cells were washed with PBS twice and then treated with 0.5 mM DSP in PBS for 10 min at room temperature. Cells were then washed with PBS twice and maintained in quenching solution containing 50 mM glycine in PBS, pH 7.4, for 5 min. Cells were subsequently lysed in ice-cold RIPA buffer (25 mM HEPES at pH 7.4, 0.1% SDS, 1% Nonidet P-40, 0.5% sodium deoxycholate, 1 mM dithiothreitol, 200 μM Na_3_VO_4_, 4 μg/mL aprotinin, 100 μM phenylmethylsulfonyl fluoride, and 2 μg/mL leupeptin). Cell lysates were gently rocked with a primary antiserum at 4°C overnight, and then incubated in 30 μL protein G-agarose (50% slurry) at 4°C for 2 h. Alternatively, the cell lysates were incubated in 30 μL anti-Flag affinity agarose gel (50% slurry) at 4°C for 4 h. Immunoprecipitates were washed with ice-cold RIPA buffer (400 μL) for four times, resuspended in 50 μl RIPA buffer and 10 μl 6× sample buffer and then boiled for 5 min. Target proteins in the immunoprecipitates were analyzed by Western blots. Signal intensities of the immunoreactive bands were quantified using Image J software, version 1.38x (National Institutes of Health, USA).

### Expression and purification of recombinant Gα_16_ and Fhit proteins, and GST pull-down

Fhit and Gα_16_ were subcloned into pGEX-4 T-1 and pET21a(+) expression vectors, respectively, and transformed into *E. coli* BL21 strain. 750 ml bacterial cultures were grown at 37°C until the OD_600_ reached 0.6-0.8. The cultures were cooled down at 4°C for 20 min and 0.2 mM IPTG was added. The cultures were then grown at 18°C overnight (for GST-Fhit) or 30°C for 15 h (for His-Gα_16_).

Cells were harvested by centrifugation for 15 min at 6,000 rpm and resuspended in 30 ml ice-cold lysis buffer for GST-tagged Fhit (50 mM Tris, pH 7.5, 500 mM NaCl, 2 mM EDTA, 1 mM dithiothreitol, 1 mM phenylmethylsufonyl fluoride, 2 μg/ml leupeptin) and lysed by three rounds of sonication. After addition of Triton X-100 to a final concentration of 1%, the lysate was incubated at 4°C for 10 min. Cell debris was removed by centrifugation at 18,000 rpm for 20 min. The cleared supernatant was then incubated with Glutathione Sepharose™ 4 Fast Flow beads at 4°C for 1.5 h with gentle rotation. The beads were spun down at 4,000 rpm for 1 min and washed four times with wash buffer (lysis buffer with 150 mM NaCl and 10% glycerol). The beads were then loaded into a chromatography column and GST-Fhit was eluted washing buffer containing 20 mM glutathione. Similar procedure was used for the purification of His-tagged Gα_16_ except that Ni-NTA Agarose and a different lysis buffer was employed (PBS, pH 8.0, 300 mM NaCl, 10 mM imidazole, 1 mM dithiothreitol, 1 mM phenylmethylsufonyl fluoride, 2 μg/ml leupeptin and 20 mM 2-mercaptoethanol). His-Gα_16_ was eluted in washing buffer containing a discontinuous gradient of imidazole (from 30 mM to 250 mM). Proteins eluted at fractions 6 and 7 were pulled. Purified GST or GST-Fhit were mixed with Gα_16_ (2 μg each) in 500 μL pull-down buffer (50 mM Tris–HCl, pH 7.5, 100 mM NaCl, 40 mM NaP_2_O_7_ and 5 mM MgCl_2_) in combination with 1 μM GDPβS or GTPγS, and then the mixture was incubated at 4°C for 30 min. Glutathione sepharose was then added and the mixture was further incubated at 4°C for 2 h. After being washed with pull-down buffer twice, the beads were resuspended in sample buffer and subjected to Western blot analysis.

### Assay for diadenosine triphosphate hydrolysis by recombinant Fhit

100 μM of Ap_3_A was incubated with or without recombinant GST-Fhit protein or GST protein in 50 mM HEPES-NaOH, pH 6.8, containing 0.5 mM MnCl_2_ for 10 min at 37°C in a total volume of 100 μl. Reactions were stopped by heat inactivation (95°C, 10 min). 50 μl of nucleotide standards and assay solutions were then analyzed by HPLC with a Mono Q column, eluted with a gradient from 50 to 600 mM ammonium bicarbonate, pH 8.5, at a flow rate of 1 ml/min. Absorbance of nucleotides were detected at 254 nm. For reactions that required His-Gα_16_ incubations, 0.5 μg His-Gα_16_ was pre-incubated with either GDPβS or GTPγS (100 μM each) at 30°C for 30 min in GTP binding activation buffer (50 mM Hepes, pH 8, 10 mM MgCl_2_, 1 mM dithiothreitol, 1 mM EDTA, and 100 mM NaCl) prior to incubation with Fhit/Ap_3_A for 10 min. The extent of Ap_3_A hydrolysis by 0.5 μg GST-Fhit was measured in the absence or presence of His-Gα_16_, and was expressed as percentage of Ap_3_A hydrolyzed during the reaction based on the areas under the peaks of Ap_3_A before and after the hydrolysis reaction [38].

### Ras activation assay

HEK293 cells were co-transfected with 200 ng Gα, 200 ng Flag-Fhit and 100 ng Ras cDNAs. After 1 day, transfectants were serum starved for 4 h. Cells were then washed twice with ice-cold PBS and lysed with the Mg^2+^lysis buffer (MLB; 125 mM HEPES at pH 7.5, 750 mM NaCl, 5% Nonidet P-40, 50 mM MgCl_2_, 5 mM EDTA, 10% glycerol, and appropriate protease inhibitors). Clarified cell lysates were immunoprecipitated with 20 μL Raf-1 RBD agarose for 45 min and subsequently washed three times with 400 μL ice-cold MLB. Eluted protein samples in 50 μL MLB and 10 μL 6× sampling dye were then resolved in SDS gels and analyzed using specific anti-Ras antibody.

### Inositol phosphates accumulation assay

HEK293 cells were seeded on a 12-well plate at 2 × 10^5^ cells/well one day prior to transfection. Various cDNAs at a concentration of 0.5 μg/well were transiently transfected into the cells using Lipofectamine PLUS® reagents. One day after transfection, cells were labeled with inositol-free Dubecco’s modified Eagle’s medium (DMEM; 750 μL) containing 5% FBS and 2.5 μCi/mL *myo*-[^3^H]inositol overnight. The labeled cells were then washed once with IP_3_ assay medium (20 mM HEPES, 5 mM LiCl, serum-free DMEM) and then incubated with 500 μl IP_3_ assay medium at 37°C for 1 h. Reactions were stopped by replacing the assay medium with 750 μL ice-cold 20 mM formic acid and the lysates were kept in 4°C for 30 min before the separation of [^3^H]inositol phosphates from other labeled species by sequential ion-exchange chromatography as described previously [56].

### Transfection of HeLa cells with Fhit siRNA

Previously validated siRNA against Fhit (Fhit si1 sequence; [57]) was used for the knockdown of Fhit. HeLa cells (1 × 10^6^ cells) cultured in 10-cm plates were transfected with siFhit (50 nM; Ribobio, Guangzhou, China) or a negative universal control med GC siRNA by Lipofectamine^TM^ RNAiMAX (Invitrogen, Carlsbad, CA). After 24 h incubation, 2 × 10^4^ cells per well were seeded into 96-well plates for Ca^2+^ measurement or 5 × 10^5^ cells per well into 6-well plates and lysed for Western blotting.

### Western blotting analysis

Protein samples were resolved on 12% SDS-polyacrylamide gels and transferred to Osmonics nitrocellulose membrane. Resolved proteins were detected by their specific primary antibodies and horseradish peroxidase-conjugated secondary antisera. The immunoblots were visualized by chemiluminescence with the ECL kit from Amersham, and the images detected in X-ray films were quantified by densitometric scanning using the Eagle Eye II still video system (Stratagene, La Jolla, CA, USA).

### Measurement of intracellular Ca^2+^ by FLIPR

The intracellular Ca^2+^ was measured by using an optimized Fluorometric Imaging Plate Reader (FLIPR) protocol [58]. HeLa cells were seeded into clear-bottomed black-walled 96-well plates. The growth medium was replaced by 200 μL labeling medium containing 1:1 (v/v) ATCC-MEM medium: Hank’s balanced salt solution, 2.5% (v/v) fetal calf serum, 20 mmol/L HEPES, pH 7.4, 2.5 mmol/L probenecid and 2 μmol/L Fluo-4 AM. Histamine was prepared as a 5× solution in Hank’s balanced salt solution into another polypropylene 96-well plate. After 1 h labeling, cell and drug plates were placed in a FLIPR (Molecular Devices, Sunnyvale, CA, USA). Immediately after the addition of 50 μL of drug solution into the cell medium, changes in fluorescence were monitored over 120 s following excitation at a wavelength of 488 nm and detection at 510–560 nm.

### Luciferase assay

The growth medium of serum-starved transfectants was removed and replaced by 25 μl of lysis buffer provided in the Luciferase Reporter Gene Assay kit (Roche Applied Science). The 96-well microplate was shaken on ice for 30 min. The luciferase activity was determined by a microplate luminometer LB96V (EG&G Berthold, Germany). Injector M connected to lysis buffer and injector P connected to the luciferin substrate were set to inject 25 μl of each component into each well. A 1.6 sec delay time followed by a 2 sec measuring time period was assigned to infector M whereas injector P was measured for 10 s after introduction of luciferin into the well. Results were collected by WinGlow version 1.24 and expressed as relative luminescent units (RLU). Statistical calculation was performed using KyPlot version 2.0.

### Establishment of stable cell lines

HEK293 or H1299 cells stably expressing Flag-tagged Fhit, or the pFlag-CMV2 vector were established by LipofectAMINE-mediated transfection along with excess pcDNA3 (9:1 ratio), followed by G418 selection for 2 weeks. The resultant cell lines were named as 293/Fhit and 293/vector, or H1299/Fhit and H1299/vector, respectively.

### 3-(4,5-dimethylthiazol-2-yl)-2,5-dephenyl-tetrazolium bromide (MTT) colorimetric assay

Cells (5,000 cells/well of HEK293 or 2,000 cells/well of H1299) were seeded in 96-well plates and incubated in the absence or presence of agonists (100 μM carbachol or 100 nM bombesin) for various durations. After removing the growth medium, 100 μl MTT labeling reagent (0.5 mg/ml; Roche Applied Science) in serum-free medium was added. The plate was incubated for 4 h at 37°C prior to the addition of 100 μl solubilization buffer (10% SDS in 0.01 M HCl). The plate was incubated overnight at 37°C. The absorbance reading was taken at the wavelength of 570 nm, with the reference value taken at the wavelength of 630 nm.

## Abbreviations

PLCβ: Phospholipase Cβ; IP3: Inositol 1,4,5-trisphosphate; Ap3A: Diadenosine 5′,5′′′-P1,P3-triphosphate; GDPβS: Guanosine 5′-O-(2-thiodiphosphate); GTPγS: Guanosine 5′-O-(3-thiotriphosphate); Raf-1 RBD: Ras-binding domain of Raf-1; RGS: Regulator of G protein signaling; TPR1: Tetratricopeptide repeat 1; IP: Immunoprecipitation; MTT: 3-(4,5-dimethylthiazol-2-yl)-2,5-dephenyl-tetrazolium bromide.

## Competing interests

The authors declare that they have no competing interests.

## Authors’ contributions

HZ and GPWC performed the experiments, participated equally in the design of the study and wrote the manuscript. JZ and WWSY carried out some of the experiments. HA raised and characterized the anti-phospho-Fhit Tyr^114^ antiserum. ASLC participated in the design of experiments. YHW participated in the design of the study and the writing of the manuscript. All authors read and approved the final manuscript.

## Supplementary Material

Additional file 1 Fhit expression is increased by activated Gaq. Click here for file

Additional file 2 **The structures of active and inactive Gα**_**q**_**.** Click here for file

Additional file 3 **Co-expression of constitutively activated mutant of Gα**_**q**_** increases the stability of Fhit truncation mutants.** Click here for file

Additional file 4 Expression of endogenous Fhit in HeLa cells. Click here for file
