# Supplementary material for: Activation state-dependent interaction between Gαq subunits and the Fhit tumor suppressor
Source: Cell Commun Signal. 2013 Aug 15;11:59. doi: 10.1186/1478-811X-11-59 (PMC3751744; doi:10.1186/1478-811X-11-59)
Supplement: Additional file 1 — Fhit expression is increased by activated Gaq. [file 1478-811X-11-59-S1.pdf]

Additional File 1

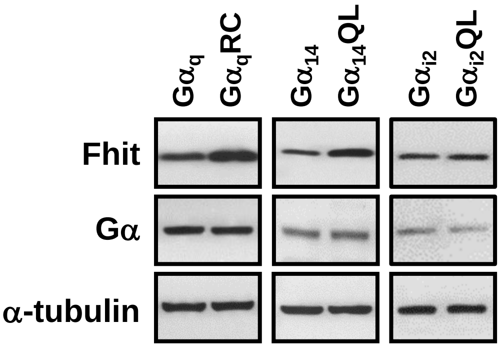

Fhit expression is inreased by activated $G\alpha_q$. HEK293 cells were co-transfected with equivalent amounts of pFlag-CMV2-Fhit in combination with a wild-type Gα subunit (pcDNA1-$G\alpha_q$, pcDNA3-$G\alpha_{14}$ or pcDNA3-$G\alpha_{i2}$), or a constitutively active Gα mutant ($G\alpha_q$RC, $G\alpha_{14}$QL or $G\alpha_{i2}$QL). One day later, cell lysates were prepared and the expression of Fhit and Gα subunits was analyzed by Western blotting with specific antisera. Overexpression of the activated members of $G\alpha_q$ subfamily ($G\alpha_q$RC and $G\alpha_{14}$QL) but not their corresponding wild-types or a member of another G protein subfamily ($G\alpha_{i2}$ and $G\alpha_{i2}$QL) could increase Fhit expression in HEK293 cells.
