# Supplementary material for: Activation state-dependent interaction between Gαq subunits and the Fhit tumor suppressor
Source: Cell Commun Signal. 2013 Aug 15;11:59. doi: 10.1186/1478-811X-11-59 (PMC3751744; doi:10.1186/1478-811X-11-59)
Supplement: Additional file 2 — The structures of active and inactive Gαq. [file 1478-811X-11-59-S2.pdf]

Additional File 2

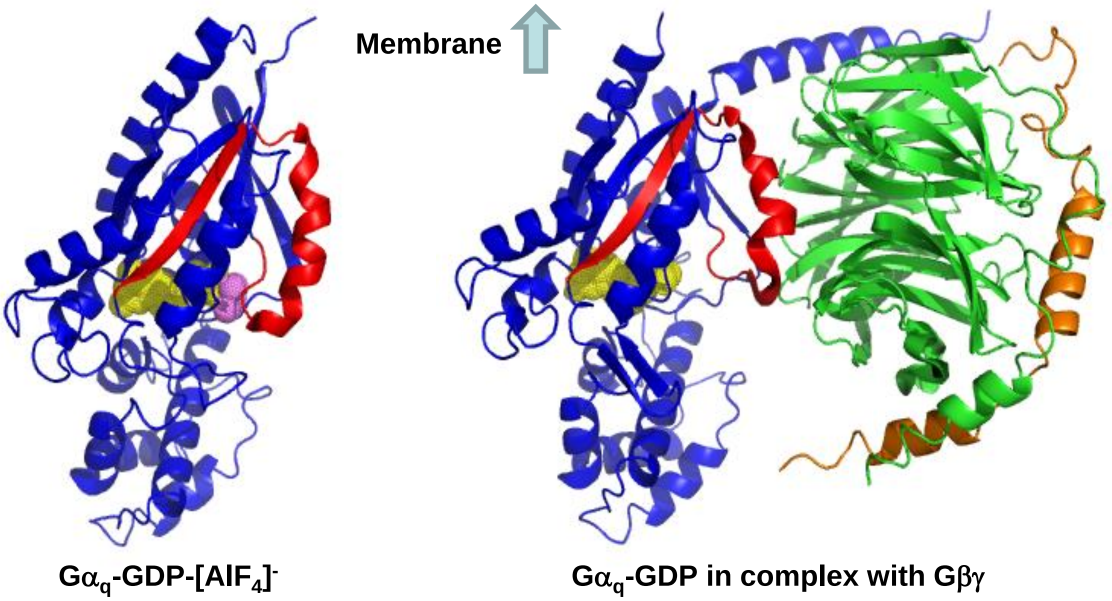

The structures of active and inactive Gα$_q$. Molecular modeling of Gα$_{16}$ based on the crystal structure of active Gα$_q$ [left, in a complex with p63RhoGEF and RhoA, PDB ID: 2RGN_A] or inactive Gα$_q$ with Gβγ complex [right, PDB ID: 3AH8] to illustrate the location of the α2-β4 regions (red) relative to the other domains (blue). The relative positions of the Gβ subunit (green), Gγ subunit (orange), GDP (yellow), and [AlF$_4$]$^-$ (purple) are also illustrated. Arrow indicates the topology of the plasma membrane. Based on the structures, molecular modeling of Gα$_{16}$ predicted that the α2-β4 domain interacts with Gβγ in the inactive state but becomes exposed to the outer surface in the active state.
