# Supplementary material for: Activation state-dependent interaction between Gαq subunits and the Fhit tumor suppressor
Source: Cell Commun Signal. 2013 Aug 15;11:59. doi: 10.1186/1478-811X-11-59 (PMC3751744; doi:10.1186/1478-811X-11-59)
Supplement: Additional file 3 — Co-expression of constitutively activated mutant of Gαq increases the stability of Fhit truncation mutants. [file 1478-811X-11-59-S3.pdf]

Additional File 3

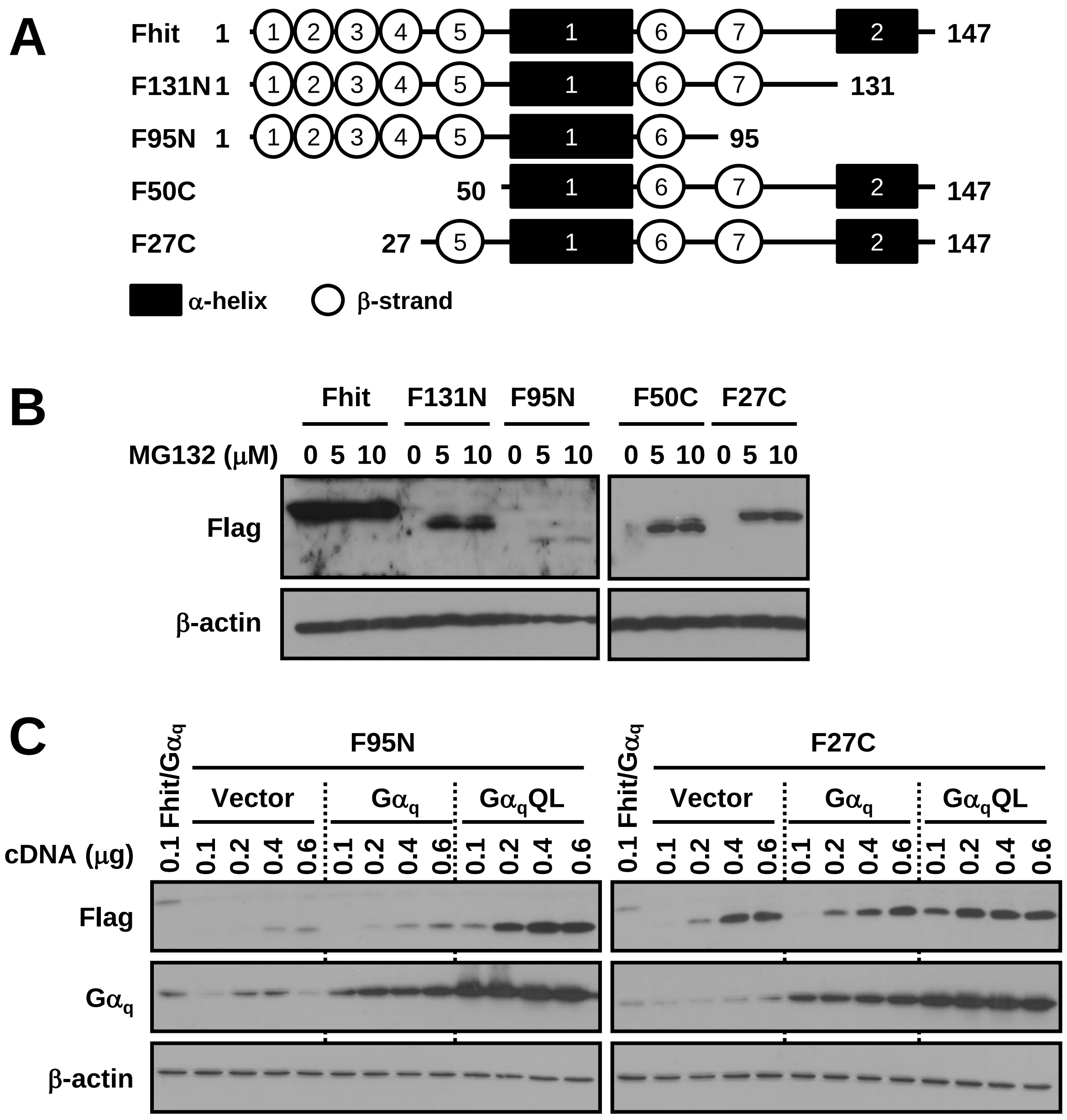

Co-expression of constitutively activated mutant of $G\alpha_q$ increased the stability of Fhit truncation mutants. *A*, Schematic representation of the Fhit truncation mutants, F131N, F95N, F50C and F27C. Predicted secondary structures are illustrated as filled boxes (α helices) or open ovals (β strands) above the mutants. *B*, The cDNAs of wild-type Fhit and Fhit truncation mutants were transfected into HEK293 cells and one day later the cells were treated with 0, 5 or 10 μM MG132 (a proteasome inhibitor). The expression levels of wild-type Fhit and Fhit truncation mutants were detected by Western blotting. It was difficult to detect the expression of Fhit truncation mutants unless the protein degradation through the proteasome was inhibited. Deletion at either terminus of Fhit results in the rapid degradation of the mutants. *C*, HEK293 cells were seeded into 6-well plates and 0.1, 0.2, 0.4 or 0.6 μg of F95N or F27C cDNA was co-transfected with pcDNA3 (Vector), $G\alpha_q$, or $G\alpha_q$QL. As a control, 0.1 μg wild-type Fhit cDNA was co-transfected with $G\alpha_q$ for comparison. Expressions of target proteins were analyzed by Western blotting. The expressions of F95N and F27C were up-regulated by the co-expression of $G\alpha_q$QL, while the co-expression of $G\alpha_q$ had no such effect on Fhit expression as compared to the vector co-expression. The extensive degradation of the truncation mutants and their uneven expressions rendered co-immunoprecipitation assays extremely hard to interpret.
