# Supplementary material for: Activation state-dependent interaction between Gαq subunits and the Fhit tumor suppressor
Source: Cell Commun Signal. 2013 Aug 15;11:59. doi: 10.1186/1478-811X-11-59 (PMC3751744; doi:10.1186/1478-811X-11-59)
Supplement: Additional file 4 — Expression of endogenous Fhit in HeLa cells. [file 1478-811X-11-59-S4.pdf]

**Additional File 4**

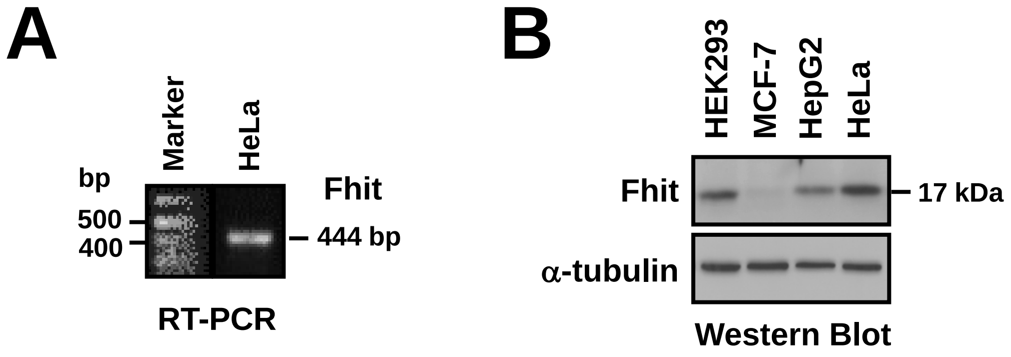


**Normal Fhit was expressed in HeLa cells.** *A*, Fhit mRNA in HeLa cells were detected by RT-PCR. Primers of Fhit (5’-atgtcgttcagatttggc-3’ and 5’-tcactgaaagtagacccg-3’) were used to examine full-length mature Fhit mRNA (444 bases). A band corresponding to the predicted size of the Fhit transcript was obtained from the total RNA of HeLa cells. *B*, Cell lysates were prepared from HEK293, MCF-7, HepG2, and HeLa cells and 50 μg protein of each sample was loaded to the SDS-PAGE gel. Detection by anti-tubulin was used as a loading control. Endogenous Fhit (17 kDa) levels were detected by an anti-Fhit antisera. HEK293, HepG2, and HeLa cells were Fhit-positive, while MCF-7 cells were Fhit-negative.
